# Editorial: Plant Responses to Phytophagous Mites/Thrips and Search for Resistance

**DOI:** 10.3389/fpls.2019.00866

**Published:** 2019-07-04

**Authors:** Raul A. Sperotto, Vojislava Grbic, Maria L. Pappas, Kirsten A. Leiss, Merijn R. Kant, Calum R. Wilson, M. Estrella Santamaria, Yulin Gao

**Affiliations:** ^1^Graduate Program in Biotechnology, University of Taquari Valley–Univates, Lajeado, Brazil; ^2^Department of Biology, University of Western Ontario, London, ON, Canada; ^3^Department of Agricultural Development, Democritus University of Thrace, Orestiada, Greece; ^4^Horticulture, Wageningen University & Research, Wageningen, Netherlands; ^5^Department of Evolutionary and Population Biology, Institute for Biodiversity and Ecosystem Dynamics, University of Amsterdam, Amsterdam, Netherlands; ^6^Tasmanian Institute of Agriculture, University of Tasmania, Hobart, TAS, Australia; ^7^Centro de Biotecnología y Genómica de Plantas, Instituto Nacional de Investigación y Tecnología Agraria y Alimentaria, Universidad Politécnica de Madrid, Madrid, Spain; ^8^State Key Laboratory for Biology of Plant Diseases and Insect Pests, Institute of Plant Protection, Chinese Academy of Agricultural Sciences, Beijing, China

**Keywords:** mites, thrips, plant responses, defense, resistance, tolerance

Phytophagous mites and thrips are global pests affecting a wide range of agricultural crops (Mouden et al., 2017; Agut et al., 2018). Among the arthropods, they are phylogenetically distant, but both classes harbor species ranging from highly specialized to extremely polyphagous (Rioja et al., 2017; Wu et al., 2018). Through convergent evolution, mites and thrips evolved stylets to facilitate feeding from mesophyll or epidermal cells (Bensoussan et al., 2016; Rioja et al., 2017; Wu et al., 2018). Despite large crops losses (Agut et al., 2018; Steenbergen et al., 2018) that are expected to become more severe with global warming (Ximenez-Embún et al., 2017; Urbaneja-Bernat et al., 2019), the interactions between mites/thrips and their host plants have been understudied. Hence, understanding how plants defend themselves against these pests is essential for developing crop protection strategies. This Research Topic provides an update on recent advances in the plant molecular and physiological mechanisms associated with phytophagous mite/thrips-plant interactions, and provides an overview of different approaches for improving crop resistance sustainably, either through repellence, feeding disruption or prevention of feeding damage. Here, we highlight some of the major points arising from these reports.

## Mite-Related

### Plant Resistance

The ability of zoophytophagous predators such as the mirid *Macrolophys pygmaeus* to induce plant defenses and indirectly affect spider mites is well-known (Pappas et al., 2015; Zhang et al., 2018). Recently, Cruz-Miralles et al. (2019) showed that *Euseius stipulatus*, a zoophytophagous phytoseiid common in citrus, can trigger plant-genotype specific defensive responses and affect their prey beyond predation through plant-mediated effects. In this topic, Pérez-Hedo et al. report reduced infestation of tomato plants exposed to the mirid predator *Nesidiocoris tenuis* by the spider mite *Tetranychus urticae*. This effect correlated with the upregulated expression of a jasmonic acid-responsive gene (*PIN2*) and two protease inhibitors markers (*PI-II1* and *PI-II2*) in tomato plants, indicating that crop protective benefits of zoophytophagous predators include both herbivore predation and the induction of plant innate defenses. Furthermore, the negative correlation of the zingiberene content of tomato trichomes and spider mite performance was described by de Oliveira et al. demonstrating a multifaceted role of this terpene in defense response. Glandular trichomes and zingiberene contribute greatly to the resistance of wild tomatoes against herbivores (Glas et al., 2012). The ability to accumulate zingiberene in glandular trichomes is being transferred to commercial varieties to increase resistance to mites and whiteflies (Bleeker et al., 2012).

The red spider mite, *Tetranychus neocaledonicus*, can be an important pest on lima bean (*Phaseolus lunatus*) (Gomes Neto et al., 2017). de França et al. assessed antibiosis and antixenosis effects of nine lima bean genotypes against the red spider mite and identified two distinct groups. The authors propose that one set of these genotypes can be used in trap cropping strategies, and the second can be used as a source of resistance against *T. neocaledonicus*.

*T. urticae* genome contains a large number of cysteine- and serine-proteases, indicating their importance in spider mite physiology (Santamaria et al., 2015). However, cystatins and serine-protease inhibitors are plant defense proteins that target these mite proteins (Santamaria et al., 2012; Martel et al., 2015). Arnaiz et al. investigated the role of Arabidopsis Kunitz Trypsin Inhibitors (KTI) on plant defense against spider mite and showed that KTI confer resistance to *T. urticae*. Moreover, transient overexpression of *KTI4* and *KTI5* in tobacco demonstrated their bifunctional ability to inhibit cysteine- and serine-proteases. It is expected that KTI impact mite gut cysteine-proteases involved in the hydrolysis of dietary proteins and can thus be used as a potential tool to engineer plant resistance/tolerance to *T. urticae*.

Monocots, including grasses, are attacked by generalist and specialist herbivores. Several spider mite species are key pests on cereals, including the generalist *T. urticae* (Grbić et al., 2011) and the Poaceae specialist *Oligonychus pratensis* (Bynum et al., 2015). Bui et al. provided evidence that maize and barley defenses induced by *T. urticae* and *O. pratensis* herbivory are similar. However, a functional benzoxazinoid pathway negatively affected only the performance of the generalist *T. urticae* and not the specialist *O. pratensis*, suggesting that *O. pratensis* adapted to the maize as a plant-host by evading benzoxazinoid defenses. Similarly, Arena et al. used RNAseq to assess the global response of Arabidopsis upon the infestation of the false spider mite *Brevipalpus yothersi*. *Brevipalpus* feeding induced jasmonic acid (JA)- and salicylic acid (SA)-regulated Arabidopsis responses, very similar to those resulting from *T. urticae* feeding (Zhurov et al., 2014). However, Arena et al. demonstrated that Arabidopsis defenses affect *Brevipalpus* differently than *T. urticae*, since *Brevipalpus* is insensitive to JA-regulated defenses, and requires the SA pathway for maximal performance. The comparative analysis of mechanisms underlying differential responses of *T. urticae, O. pratensis*, and *Brevipalpus* to plant defenses will provide new insights into mechanisms of mite adaptation to different host plants.

The phytohormones gibberellic acid (GA) and JA regulate plant growth/development and defense, respectively (Hou et al., 2013). Several studies have addressed the plant dilemma between “to grow” and “to defend” in response to various stimuli, indicating that plants prioritize GA- or JA-induced responses (Heinrich et al., 2013; Qin et al., 2013; De Bruyne et al., 2014; Wang et al., 2015; Campos et al., 2016; Guo et al., 2018). Sperotto et al. observed that wild rice species are highly susceptible to the mite *Schizotetranychus oryzae*. This was possibly due to a high GA:JA ratio, since the tested wild species are relatively tall (1.5–5 m). Therefore, authors suggest the use of short and stocky *Oryza* species as primary sources of mite resistance.

While resistance is aimed at maximizing plant fitness by targeting herbivores, other traits may maximize fitness through compensatory physiological responses (Koch et al., 2016; Erb, 2018), while tolerating the herbivore. Very little is known about the genetic mechanisms of tolerance (Peterson et al., 2017). In an Opinion article, Sperotto et al. reason that crops are tolerant when they produce acceptable yields and maintain fitness when infested. By evaluating rice morphology and productivity, Buffon et al. subsequently identified a rice cultivar tolerant to *S. oryzae*. Together they argue that tolerance, as a mechanism for maximizing yield and productivity, should be more at the forefront of crop protection research, since this is what really matters to farmers.

### Responses to Mixed Stimuli

Plants are exposed to numerous biotic and abiotic stresses. To survive these challenges they have to undergo distinct physiological and structural transformations that can be costly (Wang et al., 2003). Plant responses to abiotic stress may also affect the performance of herbivores (Scheirs et al., 2006). Some plants evolved the ability to accumulate heavy metals from the soil in their shoots. It was suggested that this can provide protection against herbivores (Boyd, 2007; Hörger et al., 2013). Godinho et al. showed that tomato plants can accumulate cadmium (Cd) to levels that negatively affect spider mite performance, while not affecting the plant. They observed detrimental Cd effects on mites, regardless of their ability to induce or suppress plant defenses. This suggests that Cd-accumulation could provide a plastic plant resistance trait that can also counteract defense-suppression by herbivores. Furthermore, the study of *Tetranychus evansi* performance on drought-adapted tomatoes showed a positive link between the induction of soluble carbohydrates and amino acids used by the plant for osmotic adjustment during drought and mite performance (Ximénez-Embún et al.). *Tetranychus evansi* downregulated the accumulation of defense-related phytohormones in control and drought-adapted plants, indicating that drought promotes *T. evansi* tomato infestation.

Beneficial microorganisms are known to promote plant growth and confer resistance to biotic and abiotic stressors (Pieterse et al., 2014; Finkel et al., 2017). Pappas et al. studied the effect of a beneficial endophytic fungus (*Fusarium solani*, FsK) on tomato defenses against *T. urticae*, and showed that both direct and indirect defenses can be enhanced on endophyte-colonized plants. Defense-related genes were differentially expressed and *T. urticae* performance was negatively affected by FsK-colonization. Furthermore, FsK-colonized plants emitted different volatiles in response to *T. urticae* compared to control plants, and were more attractive to *Macrolophus pygmaeus*, a natural enemy of spider mites. Therefore, three-way interactions (such as tomato-FsK-*T. urticae*) may offer the opportunity for the development of novel tools for spider mite control.

Wheat curl mite (WCM), *Aceria tosichella*, is a major pest of wheat that vectors damaging plant viruses (Navia et al., 2013). Skoracka et al. reviewed the current knowledge on WCM-wheat-virus interactions and identified gaps in underlying mechanisms of mite infestation, viral epidemiology, and plant responses. They emphasize the application of molecular techniques in mite-wheat-virus studies and discuss the possibilities for breeding cereal cultivars carrying resistance genes against WCM and viruses.

### Modulation of Plant Defense Response

Some herbivores evolved the ability to counteract plant defenses by producing effectors that disrupt plant signaling and induce effector-triggered susceptibility (Hogenhout and Bos, 2011; Ferrari et al., 2013; Pel and Pieterse, 2013). Accordingly, several species of spider mites were shown to suppress plant defenses (Kant et al., 2008) via effectors (Villarroel et al., 2016). While under laboratory conditions this can promote mite performance, it can also encourage competition and predation (Ataide et al., 2016). Blaazer et al. asked why the suppression trait is common among mites and argue that buffering traits may shield it from natural selection. Thus, mites in nature may have to work hard to limit the ecological costs associated with suppression and to keep a monopoly on their feeding site.

### Others (Digestive System of *Tetranychus urticae* and Eriophyoid Mites)

Plants evolved several strategies to deter herbivory. These traits, in turn, have selected for counter-adaptations in herbviores to cope with plant defenses (Heidel-Fischer and Vogel, 2015). The wide host range of *T. urticae* suggests that it may have evolved general traits that allow digestion and detoxification of a wide range of different plant compounds (Rioja et al., 2017). Bensoussan et al. described the organization and properties of *T. urticae* alimentary system, as well as the functional properties of digestive compartments relative to their ability to parcel out molecules of different weights. Together with genomic and reverse genetics tools, this will enable a functional dissection of the *T. urticae* gut to identify the specific features that enabled the evolution of *T. urticae* extreme generalist feeding strategy.

Eriophyoid mites are extremely small phytophagous arthropods with unusual morphological, biological and behavioral specialization compared to other Acari (Skoracka et al., 2010). Many of them are major plant pests, and some increase their impact by transmitting plant viruses (Stenger et al., 2016; Skoracka et al.). De Lillo et al. reviewed current knowledge on agriculturally relevant eriophyoids with emphasis on sources for host plant resistance. This review aims to guide future efforts for achieving basic, specific, and applied goals in plant protection against these mites.

## Thrips-Related

### Plant Resistance

Constitutive resistance to thrips across all plant life stages is essential for more sustainable and successful cultivation of *Capsicum* (Ssemwogerere et al., 2013). Visschers et al. screened 40 *Capsicum* accessions for resistance to *Frankienella occidentalis* and *Thrips tabaci* over the plant's ontogenetic development by measuring leaf damage. Results show that resistance in *Capsicum* is species-specific and its levels determined by the plant's developmental stage, suggesting that breeding for resistance should not rely on screening in only one ontogenetic stage.

Thrips are also major pests of peanut worldwide and serve as vectors of devastating orthotospoviruses (Riley et al., 2011). Srinivasan et al. describe field resistance to different thrips species in peanut based on morphological or chemical traits. They also discuss screening methods, marker-assisted selection and genetic modifications that can be integrated to manage thrips and associated viruses, and layout future directions in peanut thrips management.

Some thrips species can induce gall formation, thereby altering the development of host tissues (Hori, 1992). Galls are obtained via manipulation of the host's cellular communication system and often include suppression of defenses (Oates et al., 2016). Jorge et al. studied structural and chemical changes in *Myrcia splendens* plants associated with galls induced by *Nexothrips* sp. Major structural changes during gall formation included alteration of the number and size of oil glands, which could affect leaf volatile production. Comparing the headspace volatiles, over 80 different compounds were differentially detected. It was concluded that presence of methyl salicylate in non-galled samples may be a bioindicator for host resistance.

### Responses to Mixed Stimuli (Induced Resistance Against Thrips)

Fungal endophytes may prime plant defenses resulting in a stronger and/or faster response to attack by herbivores (Brotman et al., 2010). Muvea et al. demonstrated that colonization with the endophyte *Hypocrea lixii* plays an important role in mediating induced resistance to *Thrips tabaci* in onion. Plants colonized with the endophyte showed substantially decreased thrips feeding activity, as well as reduced replication of Iris yellow spot virus (IYSV), resulting in decreased disease incidence.

Inducible plant defense against western flower thrips (WFT) have recently been proposed as a promising tool for thrips control (Steenbergen et al., 2018). Chen et al. showed that *Pseudomonas syringae* pv. tomato DC3000 (Pst) activates the JA signaling pathway through the production of phytotoxin coronatine (COR). Infection of tomato plants with non-pathogenic concentrations of Pst or spray treatments with COR led to significant reduction of WFT feeding damage.

### New Technologies for Recording and Analysis of Insect Behavior

The search for genetic resistance against thrips has been ongoing for a long time (Douglas, 2018). However, the toolbox for studying genetic resistance to thrips is limited. Abd-El-Haliem et al. present a method for the identification of genes relevant to thrips performance. They utilized an *Agrobacterium tumefaciens*-mediated expression of candidate thrips genes within leaf discs of the target host on which subsequently thrips performance was assessed. The methodology allows for testing of multiple candidate genes without the need for production of stable transformed plants.

Host-plant resistance to insects, like thrips, is a complex trait difficult to phenotype quickly and reliably. A crucial element is the accurate estimation of resistance level, which requires robust phenotyping systems that can accurately screen many different plant lines in a high-throughput manner (Kloth et al., 2012; Goggin et al., 2015). Jongsma et al. introduce novel hardware and software to facilitate insect choice-assays and to automate the acquisition and analysis of movement tracks. The analysis resulted in much larger contrasts in behavior traits than previously reported. Compared to leaf damage assays on whole plants or detached leaves, this method is faster and more reliable than previous methods.

## Mite/Thrips-Related

There are indications that plant responses against herbivores may be influenced by bacteria associated with herbviores (Chung et al., 2013; Su et al., 2015). Schausberger discussed the abundance and diversity of bacteria associated with spider mites and thrips, and argued that these can have a profound impact on herbivore-induced plant defense responses. Gut/saliva-associated and endosymbiotic bacteria introduced into plants during feeding can induce plant defenses, such as the activation of SA pathway, which decreases the expression of JA pathway. Schausberger stressed there is need for further research to pinpoint the effects that different bacterial groups—and their elicitors—have on plant defense against mites and thrips.

## Final Comment

In summary, the work presented here documents recent advances in the interface between plants and mites or thrips. Now, the challenge is to move forward, to integrate this information and to develop knowledge-driven sustainable control strategies against a diverse range of mite and thrips pests.

## Author Contributions

All authors listed have made a substantial, direct and intellectual contribution to the work, and approved it for publication.

### Conflict of Interest Statement

The authors declare that the research was conducted in the absence of any commercial or financial relationships that could be construed as a potential conflict of interest.
